# The mixture toxicity of heavy metals on *Photobacterium phosphoreum* and its modeling by ion characteristics-based QSAR

**DOI:** 10.1371/journal.pone.0226541

**Published:** 2019-12-19

**Authors:** Jianjun Zeng, Fen Chen, Mi Li, Ligui Wu, Huan Zhang, Xiaoming Zou

**Affiliations:** School of Life Science, Jinggangshan University, Ji’an, China; Meiji Pharmaceutical University, JAPAN

## Abstract

Organisms are frequently exposed to mixtures of heavy metals because of their persistence in the environment. The mixture toxicity of heavy metals should therefore be evaluated to perform a rational environmental risk assessment for organisms. In this study, we determined the inhibition toxicity of five heavy metals (Cu^2+^, Co^2+^, Zn^2+^, Fe^3+^ and Cr^3+^) and their binary mixtures to *Photobacterium phosphoreum* (*P*. *phosphoreum*). We obtained the following results: (1) the order of individual toxicity was Zn^2+^>Cu^2+^>Co^2+^>Cr^3+^>Fe^3+^, and (2) different combined effects (additive, synergistic and antagonistic) were observed in the binary mixtures of heavy metals, with toxicity unit (TU) values ranging from 0.15 to 3.50. To predict the mixture toxicity of heavy metals, we derived the ion characteristic parameters of heavy metal mixtures and explored the ion-characteristic-based quantitative structure–activity relationship (QSAR) model (R^2^ = 0.750, *Q*^2^ = 0.649). The developed QSAR model indicated that the mixture toxicity of heavy metals is related to the change in ionization potential ((ΔIP)^mix^), the first hydrolysis constant (log(*KOH*)^mix^) and the formation constant value ($\log K_{f}^{mix}$).

## Introduction

Organisms are typically exposed to mixtures of heavy metals because of these metals’ persistence in the environment and common use in society [1]. Thus, compared with individual toxicity results, mixture toxicity data are certainly better for performing a comprehensive evaluation of the combined toxicological effects of heavy metal mixtures upon organisms [2–3].

Most studies to date have mainly concentrated on the toxicity of single heavy metals [4], and the results have indicated that heavy metals were toxic to many organisms at certain concentrations [5]. The median effective inhibition concentrations (EC_50_) of five heavy metals on freshwater ciliated protists mostly range from 0.01 to 1.00 mg/L [6]. Heavy metal ions were shown to be severely cytotoxic to fish cell lines [7]. The increasing realization that organisms are typically exposed to mixtures of heavy metals has raised concerns about risk assessments of heavy metal mixtures [8]. The toxic effects of Zn^2+^ and Cu^2+^ are substantially higher than those expected on the basis of the additive effects of single metals [3]. However, previous mixture toxicity results were mainly based on heavy mixtures with limited numbers of components at certain concentrations. Little is known about the true mixture toxicities of heavy metals in the real environment to organisms because of the complex nature of the compositions and the high cost of these analyses [5,9]. It is therefore necessary to develop a model to predict the toxicity of heavy metal mixtures.

In the field of mixture toxicity, a number of approaches, especially the concentration addition (CA) and independent action (IA) models [10–11], have been successfully employed to predict the toxic effects of mixtures based on the effects of individual chemicals [12]. However, the interactions of heavy metal combinations exhibit mainly interactive (e.g., synergistic or antagonistic) effects and cannot be accurately predicted by the CA and IA models [4]. The biotic ligand model (BLM), a good approach for assessing the toxicity of single heavy metals, has recently been applied to predict the mixture toxicity of heavy metals [13–14]. Furthermore, the quantitative structure-activity relationship (QSAR) model has also been shown to be a valuable tool for predicting the mixture toxicity of chemicals for both non-interactive and interactive mixtures [15–16]. However, the application of the QSAR approach to heavy metal mixtures is poorly represented in the environmental toxicology literature [17].

The ions-based QSAR model is a promising method for predicting the toxicological effects of individual heavy metals and has been well demonstrated in many studies [18]. Tatara et al. [19] proved that the toxicity of single heavy metals on *Caenorhabditis elegans* can be predicted by the ion-based QSAR model. The ion-based QSAR approach was further successfully applied to predict the toxicity of individual metals on a wide range of species [20]. However, it remains unclear whether an ion-based QSAR model can be developed to predict the mixture toxicity of heavy metals. This study addresses this problem.

*P*. *phosphoreum*, a toxicity test organism, has been widely used to assess the environmental risk of chemicals by measuring the reduction of its light emission [21]. Recently, the toxic effects of heavy metals, both individually and in mixtures, have been investigated using *P*. *phosphoreum* in a variety of studies [3,22]. Hence, the objectives of this study are to (1) determine the acute (15-min exposure) toxicity to *P*. *phosphoreum* of single heavy metals and their binary mixtures at different concentrations, (2) develop a robust and predictive QSAR model based on the characteristics of heavy metal ions, and (3) reveal the possible mixture toxicity mechanism based on the developed QSAR model.

## Materials and methods

### Chemicals and cell culture

Analytical-grade pure chemicals were used as the source of metal ions, including Zn(NO_3_)_2_, Cu(NO_3_)_2_, Co(NO_3_)_2_, Fe(NO_3_)_3_, Cr(NO_3_)_3_. The heavy metals were dissolved in 3.02% NaNO_3_ [23] at a pH of 5.30 to obtain stock solutions. *P*. *phosphoreum* was selected as test organism and purchased from the Institute of Soil Science, Nanjing, PRC. The culture medium for *P*. *phosphoreum* consisted of 5 g tryptone, 5 g yeast extract, 3 g glycerin, 1 g KH_2_PO_4_, 5 g Na_2_HPO_4_, 30 g NaCl, and 1000 mL distilled water. Before each toxicity test, *P*. *phosphoreum* were inoculated from a stock culture and then grown in a fresh liquid culture medium by shaking (120 rpm/min) at 20°C for 12 h.

### Toxicity test

The toxicity test was performed in triplicate on a SpectraMax multimode plate reader (Molecular Devices, Sunnyvale, CA) with a 96-well microplate [24], and 12 concentration gradients for each of the test metal ions were arranged in the microplate as shown in S1 Fig. The 36 edge wells were filled with distilled water to prevent the edge-effect phenomenon [25]. Twenty-four wells containing no pollutants were set as the control, and the remaining 36 wells were used as the test groups. Each well was first filled with 160 μL of the test solution followed by 50 μL of inoculum. After oscillation for 1 minute for equilibrium, the microplates were kept at 20°C for 15 min. On the basis of the decrease in relative light units (RLUs), the toxic effect of heavy metals to *P*. *phosphoreum* was presented as an inhibition ratio (I), which can be calculated according to Eq 1,

$$I=\frac{L_{0}-L}{L_{0}}\times 100\%,$$ (Eq 1)

where *L*_*0*_ and *L* are the averages of the RLUs of the controls and treatments, respectively.

### Binary mixture design

The binary mixtures were designed on the basis of the observed toxicity results for the individual heavy metals (EC_50_), and the two components in binary mixtures were arranged in the following serial toxicity ratios: 1:10, 1:10^0.5^, 1:1, 10^0.5^:1, 10:1. The detailed information for test mixtures is presented in S2 Fig and S1 Table.

### Concentration-response curve fitting

The derived concentration relationship data for pollutants were fitted with a logistic model (Eq 2),

$$y=\frac{\alpha -\delta }{1+{\left(x/EC_{50}\right)}^{\beta }}+\delta ,$$ (Eq 2)

where *y* is the response of the pollutants to *P*. *phosphoreum*; *x* is the molar concentration of the individual heavy metals and of the binary mixtures; and *α*, *β* and *δ* are the derived parameters. Higher coefficients of determination (*R*^2^) and lower root-mean-square errors (*RMSE*) correspond to better fit. Based on the fitting results, the half-maximal inhibitory concentration for the tested individuals and mixtures were expressed by EC_50_ and ${\mathrm{EC}}_{{}_{50}}^{\mathrm{mix}}$, respectively.

Toxicity units (TU) were used to characterize the joint effects between heavy metals and were calculated with Eq 3,

$$\mathrm{TU}=\frac{C_{A}}{{\mathrm{EC}}_{50A}}+\frac{C_{B}}{{\mathrm{EC}}_{50B}},$$ (Eq 3)

where *C*_*A*_ and *C*_*B*_ are the concentrations of the individual pollutants in a mixture at median inhibition when tested alone, and EC_50A_ and EC_50B_ (mol/L) are the median effective inhibition concentrations of components A and B. Simple addition is defined as 1.20>TU>0.80, TU<0.80 represents synergism, and TU>1.20 indicates antagonism [26].

### Calculating the ion characteristic descriptors for heavy metal mixtures

Frequently used ion characteristic descriptors [27–28] were selected to develop the ion-characteristic-based QSAR model, and the descriptors of five individual metals (Cu^2+^, Zn^2+^, Co^2+^, Fe^3+^ and Cr^3+^) are presented in S2 Table. In the field of mixture toxicity, the parameters of mixtures are typically derived on the basis of concentrations for individual metals and the corresponding parameters of the individual metals [15]. In the case of log(*Kow*)^mix^, for example, the parameter was derived for the octanol-water partition coefficient of mixtures [29], which can be calculated on the basis of log(*Kow*) and the concentrations for individual metals. Consequently, following this well-proven approach [30], the ion-characteristic parameters for metal mixtures (*P*^*m*^) were calculated with Eq 4.

$$P^{m}=\frac{C_{A}}{C_{A}+C_{B}}\times P^{A}+\frac{C_{B}}{C_{A}+C_{B}}\times P^{B},$$ (Eq 4)

where *P*^*A*^ and *P*^*B*^ represent the ion-characteristic parameters of single metals in binary mixtures, and the molal concentration ratios for two components are expressed as *C*_*A*_/(*C*_*A*_+*C*_*B*_) and *C*_*B*_/(*C*_*A*_+*C*_*B*_); the derived parameters for test mixtures are presented in S3 Table.

### QSAR modeling

To obtain a rational QSAR model, the partial least squares (PLS) regression was performed for the determined toxicity data (−log(EC_50_) or −log(EC_50M_)) against the ion-characteristic descriptors by using Simca-S (version 6.0; Umea, Sweden). The statistical quality of the QSAR models was evaluated by *R*^2^, the standard error of estimate (*SE*), the Fisher criterion (*F*), the p-value (*P*) and the cross-validated squared correlation coefficient of the training set (*Q*^*2*^_*(cum)*_). The stability and predictive ability of the models were examined by leave-one-out (LOO) validation, and they were characterized by *R*^2^_*(ext)*_ and the cross-validated squared correlation coefficient of the external validation set (*Q*^*2*^_*(ext)*_).

## Results

### Determination of the toxicity of individual heavy metals

The effects of heavy metals on *P*. *phosphoreum* were determined. The toxicity data (-logEC_50_) and the resulting parameters are presented in Table 1. As shown in Table 1, Zn^2+^ (-logEC_50_ = 4.75) was more toxic and Fe^3+^ (-logEC_50_ = 3.64) less toxic than the other heavy metals. The order of toxicity was as follows: Zn^2+^>Cu^2+^>Co^2+^>Cr^3+^>Fe^3+^.

**Table 1:** The individual toxicity of heavy metals and the corresponding fitting parameters.

Heavy metals	Acute toxicity parameters					
	α^b^	δ^b^^)^	β^b^^)^	R^2^	RMSE	-log(EC_50_)^a^
**Cu**^**2+**^	0.034	1.000	4.183	0.977	0.005	4.47(4.38–4.52)
**Co**^**2+**^	0.036	0.960	1.637	0.995	0.001	4.43(4.35–4.48)
**Zn**^**2+**^	0.046	0.950	1.745	0.979	0.002	4.75(4.70–4.79)
**Fe**^**3+**^	0.029	0.950	2.952	0.994	0.001	3.64(3.59–3.68)
**Cr**^**3+**^	0.023	1.000	0.948	0.947	0.003	3.67(3.61–3.72)

^a^Data was presented as 95% confidence interval.

^b^ *α*, *β* and *δ* are the derived parameters by using logistic model(Eq 2).

### Determination of the toxicity of heavy metal mixtures

On the basis of the toxic effects of the individual antibiotics (*EC*_*50*_), we evaluated the toxicity of binary mixtures (-logEC_50M_) at the equitoxic levels. The toxicity data (-logEC_50M_) and the derived toxicity units of the equitoxic ratio (*TU*^equi^) are shown in Table 2. *TU*^equi^ ranged from 0.15 to 3.50, suggesting that different joint effects (addition, synergism and antagonism) occurred in the binary mixtures of heavy metals according to the criteria of *TU*^equi^ (Eq 3).

**Table 2:** The binary mixture toxicity at the equitoxic ratios and the corresponding fitting parameters.

Mixtures(A+B)	Fitting results						TU^b^
	α	δ	β	R^2^	RMSE	-log(EC_50M_)^a^	
**Fe**^**3+**^**-Co**^**2+**^	0.004	0.950	2.847	0.832	0.009	3.67 (3.58–3.71)	1.60 (1.47–1.96)
**Fe**^**3+**^**-Cr**^**3+**^	0.076	0.955	19.527	0.979	0.004	3.90 (3.84–4.00)	0.56 (0.45–0.65)
**Fe**^**3+**^**-Zn**^**2+**^	0.002	0.950	2.577	0.581	0.016	3.54 (3.47–3.63)	2.41 (1.93–2.80)
**Fe**^**3+**^**-Cu**^**2+**^	0.106	1.000	1.555	0.939	0.008	4.06 (3.99–4.22)	0.66 (0.46–0.77)
**Co**^**2+**^**-Cr**^**3+**^	0.054	1.000	2.514	0.975	0.003	4.24 (4.08–4.27)	0.45 (0.42–0.65)
**Co**^**2+**^**-Zn**^**2+**^	0.034	0.956	2.637	0.998	0.000	4.65 (4.52–4.83)	0.29 (0.15–0.31)
**Co**^**2+**^**-Cu**^**2+**^	0.063	0.963	2.996	0.989	0.003	4.59 (4.39–4.85)	0.17 (0.09–0.26)
**Cr**^**3+**^**-Zn**^**2+**^	0.051	1.000	2.294	0.965	0.005	3.98 (3.87–4.04)	0.92 (0.78–1.15)
**Cr**^**3+**^**-Cu**^**2+**^	0.008	0.950	4.917	0.987	0.003	4.49 (4.62–4.40)	0.26 (0.19–0.32)
**Zn**^**2+**^**-Cu**^**2+**^	0.031	0.950	8.636	0.958	0.006	4.66 (4.57–4.77)	0.37 (0.25–0.40)

^a^Toxicity data was expressed as mmol/L

^b^Data was presented as 95% confidence interval.

**Fig 1:**
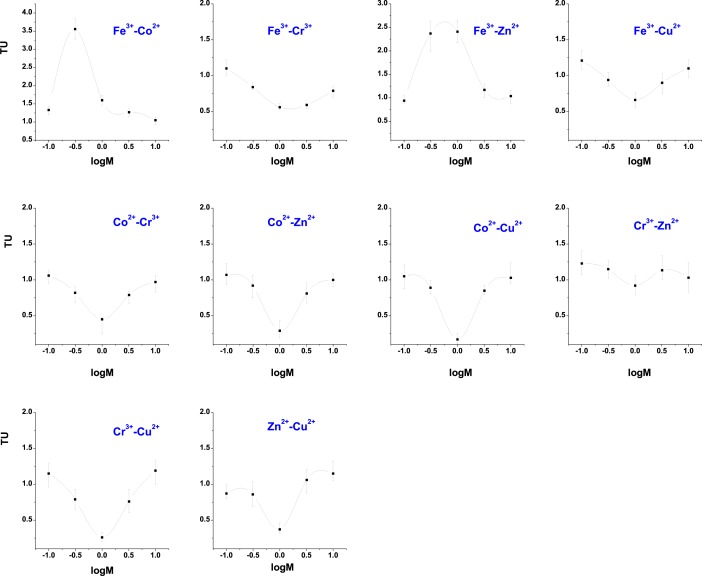
The toxicity unit (TU) of metal mixtures at non-equitoxic ratios. (logM denotes the molar concentration ratio of componets in the binary mixture).

To investigate the joint effects of heavy metals at other concentrations, we determined the toxicity of binary mixtures at non-equitoxic ratios (Fig 1). The *TU* of the mixtures were typically derived from the zone of additive action (1.20>TU>0.80), suggesting that different joint effects (addition, synergism, and antagonism) also occurred in the non-equitoxic ratio mixtures (Fig 1). As is well known, the CA and IA models [10,11] have limited ability to predict the toxic effects of non-interactive mixtures [12]. It was thus necessary to develop a model to predict the toxic effects of heavy metal mixtures in which the individual metals have joint effects.

### Developing the QSAR model

As is well documented in previous reports, metal ion-characteristic parameters can be applied to predict the toxicity of metals to organisms [20]. We therefore applied Eq 4 to calculate the ion-characteristic descriptors of heavy metal mixtures (see section 2.5 above). Eq 5 was derived on the basis of the calculated descriptors and partial least squares (PLS) regression,

$$-\log({\mathrm{EC}}_{50M})=1.639+0.113\times {\left(\Delta \mathrm{IP}\right)}^{\mathrm{mix}}+0.187\times |\log{\left(K_{OH}\right)}^{mix}|,$$ (Eq 5)

where n = 40, F = 40.234, *R*^*2*^_*(cum)*_ = 0.585, SE_*(cum)*_ = 0.212, P = 0.000, Q^2^_*(cum)*_ = 0.520; n_(ext)_ = 10, *R*^*2*^_(ext)_ = 0.515, *Q*^*2*^_(ext)_ = 0.458, SE_(ext)_ = 0.298.

R^2^_*(cum)*_ of the developed model was 0.585, indicating that (1) using *P*^*mix*^ to predict the binary mixture toxicity of metal ions is reasonable and (2) using Eq 5 to predict the binary mixture toxicity of metal ions is not feasible because of the model’s low predictive ability.

In the case of mixture toxicity, it is well accepted that the high quality QSAR model is mostly based on the toxicity mechanism [15]. Thus, we further concluded that the low predictive ability of Eq 5 resulted from an improper understanding of the toxicity mechanism of heavy metal mixtures.

According to the mixture toxicity mechanism, the toxic effects of mixtures are related to (1) their transport activities, (2) the interactions between chemicals with their protein receptors and (3) the combination of the toxic effects of the individual chemicals [31]. To examine the transport activities of heavy metal mixtures, it was assumed that the parameters of Eq 5 ((ΔIP)^mix^ and |log(*K*_*OH*_)^*mix*^|) could be applied because (1) |log(*K*_*OH*_)| denotes the first hydrolysis effect of the individual metals [28] and (2) (ΔIP) was reported to be related to the biosorption capacity(*q*_max_) of the metal ions [28].

Molecular docking is a useful approach for expressing the interactions between chemicals and their protein receptors [16]. However, the interaction between metal ions and luciferase (Luc) is difficult to investigate by molecular docking, although some heavy metal salts have been shown to inhibit Luc activity [32–33]. Fortunately, the joint effects of metal ions with firefly D-luciferin have been investigated by Riahi et al. [34] and expressed as *K*_f_ (the formation constant, Table 1). Consequently, it was assumed that if the values of *K*_f_ for the firefly were proportional to the corresponding joint effects of metal ions with Luc, a strong relationship would exist between the individual toxicity data (-log(EC_50_)) and the values of *K*_f_. Thus,

$$-\log({\mathrm{EC}}_{50})=3.483‐0.161\times K_{f}\;\mathrm{and}$$ (Eq 6)

n = 5, F = 9.479, *R*^*2*^ = 0.760, SE = 0.320, P = 0.054.

**Fig 2:**
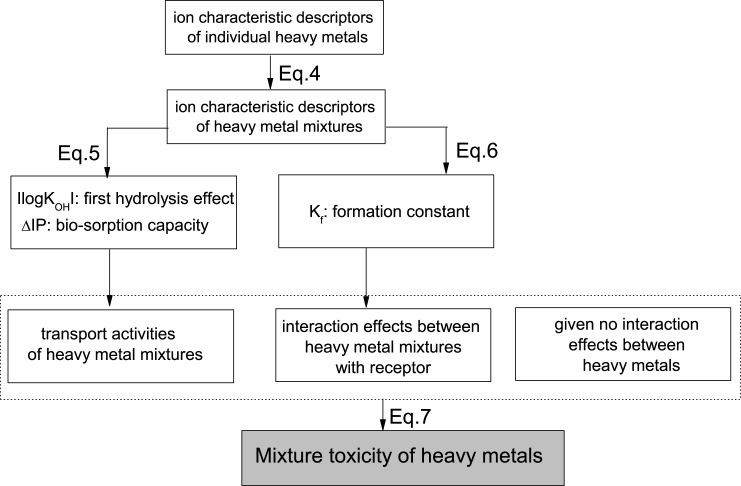
Schematic diagram illustrating the development of QSAR model for predicting the mixture toxicity of heavy metals.

As shown in Eq 6, a significant relationship (*R*^*2*^ = 0.760) exists between -log (EC_50_) and *K*_f_. This finding confirmed the assumption that, in the bioluminescence assay, the values of *K*_f_ for the firefly are proportional to the corresponding joint effects of metal ions with bacterial Luc. Consequently, based on the determined binary mixture toxicity data (S2 Table), and given that no combination toxic effects exist between heavy metals, the binary mixture toxicity of antibiotics can be reasonably characterized (Fig 2) and derived as Eq 7:

$$‐\log({\mathrm{EC}}_{50M})=1.779-0.106\times {\left(\mathrm{\Delta IP}\right)}^{\mathrm{mix}}+0.117\times |\log{\left(K_{OH}\right)}^{mix}|+0.044\times \log K_{f}^{A}+0.017\times \log K_{f}^{B};$$ (Eq 7)

n = 40, F = 26.276, *R*^*2*^_*(cum)*_ = 0.750, SE_*(cum)*_ = 0.194, P = 0.000, Q^2^_*(cum)*_ = 0.649;

n_(ext)_ = 10, *R*^*2*^_(ext)_ = 0.607, *Q*^*2*^_(ext)_ = 0.562, SE_(ext)_ = 0.204.

### Validation of the developed model

The *R*^*2*^_*(cum)*_ and *Q*^*2*^_*(cum)*_ values of the developed model (Eq 7) were 0.750 and 0.649, respectively, suggesting a good fit and that the model is robust. The *Q*^*2*^_*(Ext)*_ value of the external validation sets was 0.607, and the difference between *Q*^*2*^_*(cum)*_ and *R*^*2*^_*(cum)*_ did not exceed 0.3, indicating that the developed ion-characteristic-based model has good predictive ability and no danger of over-fitting or over-estimating the results [35].

**Fig 3:**
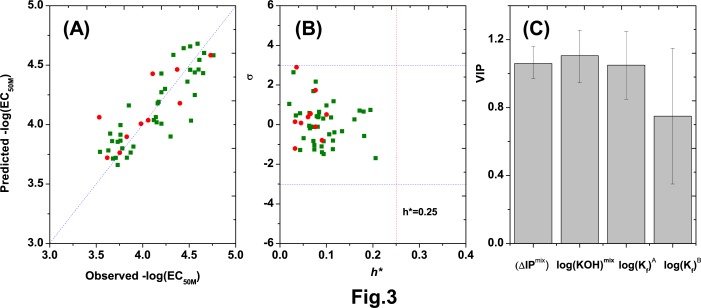
Validation and application of the developed model. (A) Plot of predicted versus observed mixture toxicities for both the training set and the validation set; (B) Williams plot showing the metal mixtures of the developed QSAR model (h* = 0.25); (C) VIP values for three variables.

The williams plot (Fig 3B) shows that there were no outliers for the response, as demonstrated by the low standardized residuals (σ) of the tests (< 3). The *h*_i_ values of all test mixtures were also lower than the h* value, suggesting that the mixtures are not influential in the mode space and that the training sets are very representative [36].

As is well known, if irrelevant or redundant variables are included in the developed model, the internal predictive power and robustness of the model will decrease [36]. VIP (variable importance in the projection), an important index that evaluates the variable importance [37], has been widely used in developing a reasonable QSAR model; the criterion (VIP value) is larger than 0.50 for important variables [38]. Therefore, we further investigated the VIP values of each variable in Eq 7. Fig 3C shows VIP values of (ΔIP)^mix^, |log(*K*_*OH*_)^*mix*^|, $\log K_{f}^{A}$ and $\log K_{f}^{B}$ of 1.06, 1.11,1.05 and 0.75, respectively, suggesting that there are no unimportant variables in the developed model. The order of VIP was |log(*K*_*OH*_)^*mix*^|>(ΔIP)^mix^>log *K*_*f*_, demonstrating that |log(*K*_*OH*_)^*mix*^| is the most sensitive parameter for predicting the mixture toxicity of heavy metals.

## Discussion

### The joint effects of heavy metal mixtures

In this study, the different joint effects (addition, synergism, and antagonism) were determined in the heavy metal mixtures (S1 Table, Fig 1) at both the non-equitoxic and equitoxic ratios. On the one hand, those results seem to be reasonable because of their good agreement with recent experimental data. The toxicity of Zn^2+^ and Cu^2+^ combinations, for example, was shown to be synergistic in some bacteria [3,39]. On the other hand, our results also indicate that the joint effects of heavy metal mixtures may differ among the test species. For instance, the toxicity of Co^2+^ and Cu^2+^ mixtures was observed to be first antagonistic and then additive or slightly synergistic for rainbow trout [40]. However, the interaction between Co^2+^ and Cu^2+^ in *P*. *phosphoreum* was synergistic in both our study and the work of Fulladosa et al. [41] and was demonstrated to be additive for earthworms [42]. Consequently, the joint effect of heavy metals is complex and not simply additive, which should be better predicted with a more rational and novel approach.

### Mechanistic implication of the developed model

Our results demonstrated that, in the developed QSAR model (Eq 7), the parameters log *K*_*f*_, |log(*K*_*OH*_)^*mix*^|, and (ΔIP)^mix^ were suitable for showing the binary mixture toxicity of heavy metals. According to Riahi [34], K_*f*_ was defined as shown in Eq 8 to express the equilibrium constant of the binding reaction (Eq 9),

$$K_{f}=\frac{\left[ML^{n+}\right]}{\left[M^{n+}\right]\left[L\right]}\times \frac{f_{\left(ML^{n+}\right)}}{f_{\left(M^{n+}\right)}f_{\left(L\right)}}$$ (Eq 8)

$$\mathrm{and}\;M^{n+}+L\overset{K_{f}}{⇄}ML^{n+},$$ (Eq 9)

where [*ML*^*n+*^], [*M*^*n+*^], [*L*] and *f* represent the equilibrium molar concentration of the complexes, the free cation, the free ligand, and the activity coefficient of the indicated species, respectively. It is obvious that increased binding of D-luciferin with the metal ions, corresponds to lower concentrations of [*M*^*n+*^] and [*L*] that can be obtained, which results in a larger K_*f*_. Consequently, a positive relationship between log *K*_*f*_ and mixture toxicity (-logEC_50M_) was observed in Eq 7.

$$K_{OH}=\frac{\left[MOH^{n-1}\right]\left[H^{+}\right]}{\left[M^{n+}\right]\left[H_{2}O\right]}$$ (Eq 10)

$$M^{n+}{+H}_{2}O\overset{K_{OH}}{⇄}{\mathrm{MOH}}^{n‐1}{+H}^{+}$$ (Eq 11)

Furthermore, log(*K*_*OH*_) is the log of the parameter for the metal’s first hydrolysis. It can be defined by Eq 10, which reflects the metal ion affinity to intermediate ligands (Eq 11) [19]. In general, log(*K*_*OH*_) is lower than zero because of the low tendency for first hydrolysis [23]. Thus, it is readily concluded that larger values of |log(*K*_*OH*_)| correspond to more [*M*^*n*+^] being supplied to bind with the receptor (Luc), which results in a higher toxicity of heavy metal mixtures. Therefore, the positive relationship between |log(*K*_*OH*_)^*mix*^| and mixture toxicity (-logEC_50M_) is also obtained in Eq 7.

Moreover, the negative relationship between (ΔIP)^mix^ and mixture toxicity (-logEC_50M_) is displayed in Eq 7. As mentioned above (Table 1), IP indicates the ionization potential, and ΔIP is the change in ionization potential. Can and Jianlong [28] derived Eq 12 to predict the biosorption capacity(*q*_max_) of metal ions. The negative relationship between ΔIP and *q*_max_ is shown in Eq 12. Because of the stronger positive relationship between *q*_max_ and the toxic effects of chemicals, the negative relationship between (ΔIP)^mix^ and mixture toxicity (-logEC_50M_) in Eq 7 is therefore reasonable.

$$q_{\max}=-0.03+0.008\times \mathrm{IP}+0.006\times (\mathrm{AN}/\Delta \mathrm{IP});$$ (Eq 12)

n = 8, F = 90.180, *R*^*2*^ = 0.990, SE = 0.009, P = 0.000;

### Comparison of this model with other models

Compared with other models, the developed QSAR model (Eq 7) provides some advantages. The first advantage lies in its application fields. As mentioned in the introduction, the ion-based QSAR model is a promising method for providing toxicological information. However, this conclusion was only demonstrated in the field of single toxicity by a number of studies. In fact, this model has greater applicability than the reported ion-characteristic-based QSAR models [20] because pollutants do not occur strictly as individual contaminants but rather as mixtures in the real environment [43]. The second advantage is the revelation of the toxicity mechanism for heavy metal mixtures. As is well known, the CA and IA models have been successfully applied to predict the toxic effects of mixtures [12], but the mixture toxicity mechanism of pollutants has typically been poorly revealed [30]. In contrast, this developed model showed that |log(*K*_*OH*_)^*mix*^| is the most sensitive parameter for predicting the mixture toxicity of heavy metals, suggesting that transport activities rather than interaction effects (log *K*_*f*_) play an important role.

Modeling necessarily has some limitations [44]. The limitations of the developed ion-characteristics-based model (Eq 7) include the following: (1) The prediction should be much better, as shown by the fact that the *R*^*2*^ of model is 0.750. This result could be due to the valence of the test metal ions. As shown by Newman et al. [17], the quality of ion-characteristics-based models for single-valent metals is typically higher than those for mixed-valent (i.e., mono-, di- or trivalent) metals. Also, the model can be more applicable if the toxic effects of other toxic metal ions (i.e. Cd) can be completely included to develop the model. (2) Limited data are available to express the interactions between Luc and the metal ions. In this study, we cited log *K*_*f*_ to show the interaction effects and our results proved that this is reasonable. However, log *K*_*f*_ was obtained from a firefly instead of *P*. *phosphoreum*; differences among species likely exist, which decreases the quality and the applicability of the developed model.

On the whole, this ion-characteristic-based model for predicting the mixture toxicity of heavy metals was first developed for mixture pollution. Because organisms are typically exposed to mixtures of heavy metals, and considering the fact that the joint effect of heavy metals is complex and not simply additive (S1 Table, Fig 1), the ion-characteristic-based QSAR approach can be potentially viewed as a supplementary tool to predict mixture toxicities of heavy metals.

## Conclusions

Different joint effects (additive, synergistic and antagonistic) occurred in the mixtures of heavy metals. According to the developed characteristic parameters of mixtures and on the basis of the mixture toxicity mechanism, a QSAR model with good fitting and prediction characteristics was first explored to predict the mixture toxicity of heavy metals. This approach permits rational environmental risk assessments of metal mixtures upon organisms.

## Supporting information

S1 Fig The setting of test groups in 96-well microplate. (DOCX)

S2 Fig The detail information of test mixtures. (DOCX)

S1 Table Used ion characteristic descriptors of test heavy metals. (DOCX)

S2 Table The information of mixtures and the corresponding parameters. (DOCX)

S3 Table The ion characteristic descriptors of mixtures that was calculated on the basis of Eq 4. (DOCX)

## Abbreviations

QSAR: quantitative structure–activity relationship
(ΔIP): change in ionization potential
log(*KOH*): first hydrolysis constant
log *K*_*f*_: formation constant value
TU: toxicity units
CA: concentration additive
IA: independent action.
