# Supplementary material for: The mixture toxicity of heavy metals on Photobacterium phosphoreum and its modeling by ion characteristics-based QSAR
Source: PLoS One. 2019 Dec 19;14(12):e0226541. doi: 10.1371/journal.pone.0226541 (PMC6922345; doi:10.1371/journal.pone.0226541)
Supplement: S1 Fig — (DOCX) [file pone.0226541.s001.docx]

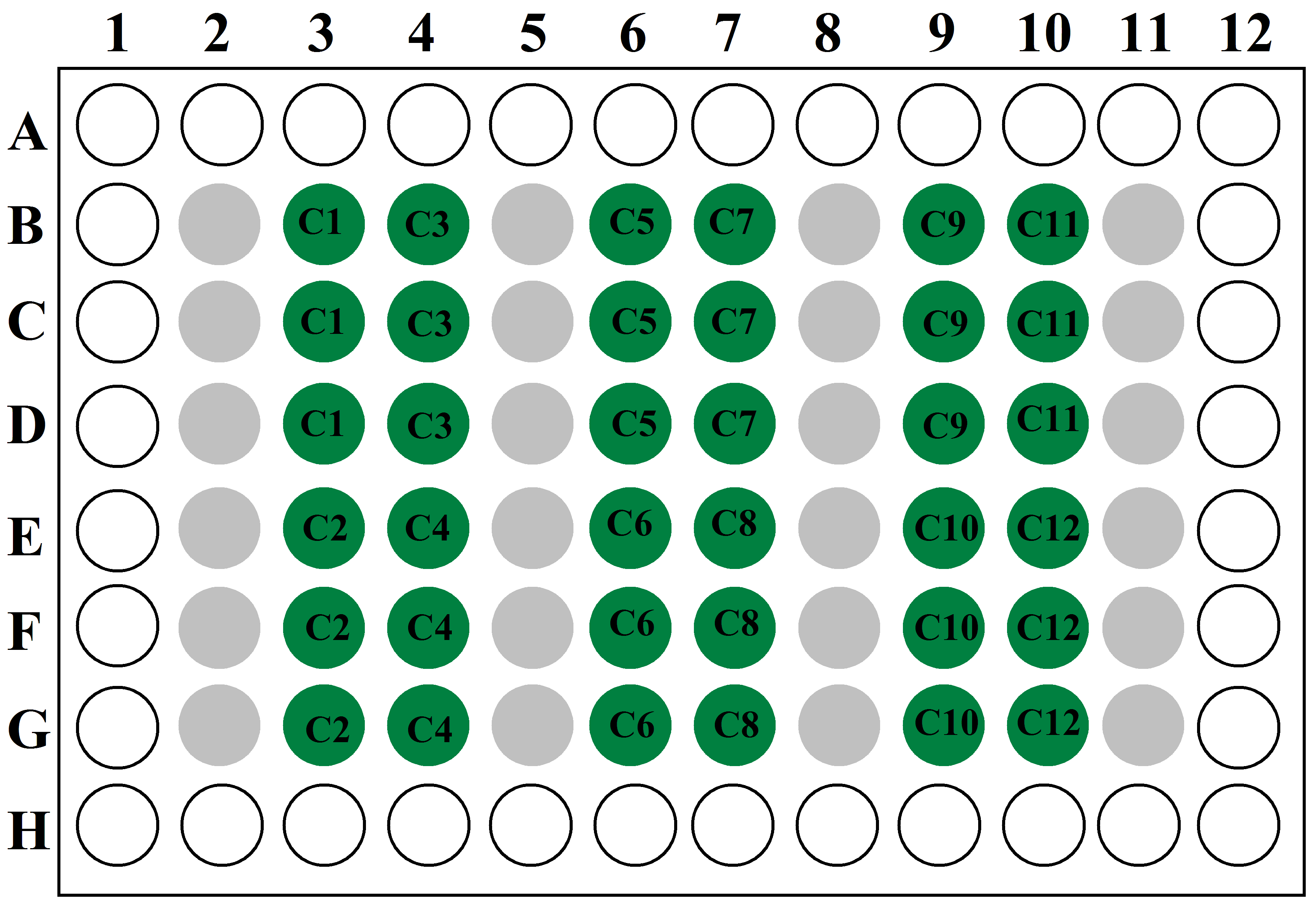


**S1Fig. The setting of test groups in 96-well microplate**.

(white circles present the edge wells; gray circles denote the control and green circles are the test wells)
