# Supplementary figures and images for: The mixture toxicity of heavy metals on Photobacterium phosphoreum and its modeling by ion characteristics-based QSAR
Source: PLoS One. 2019 Dec 19;14(12):e0226541. doi: 10.1371/journal.pone.0226541 (PMC6922345; doi:10.1371/journal.pone.0226541)

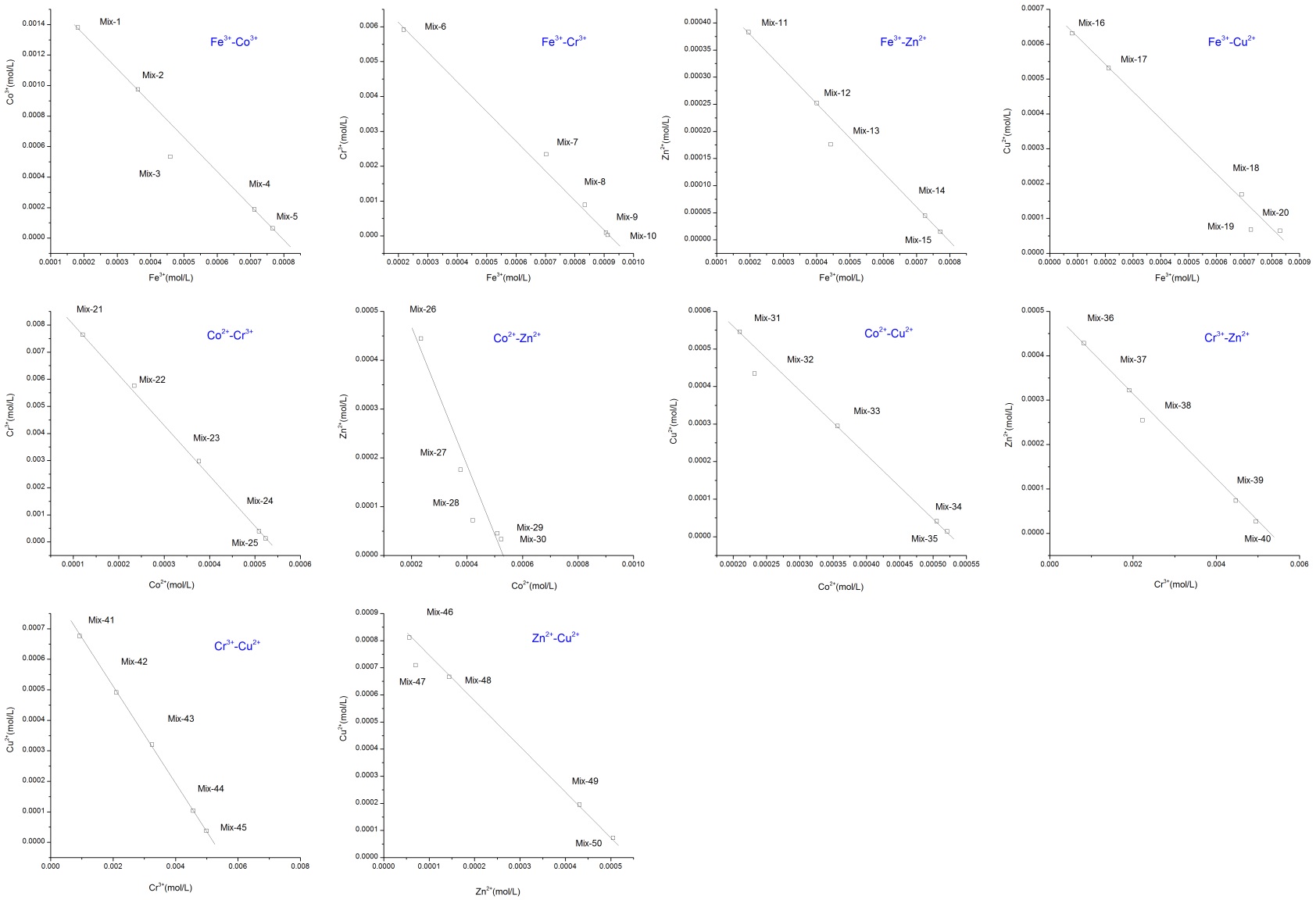


**S2 Fig. The detail information(molar concentration) of test mixtures.**

Supplement: S2 Fig — (DOCX) [file pone.0226541.s002.docx]
