# Supplementary material for: The mixture toxicity of heavy metals on Photobacterium phosphoreum and its modeling by ion characteristics-based QSAR
Source: PLoS One. 2019 Dec 19;14(12):e0226541. doi: 10.1371/journal.pone.0226541 (PMC6922345; doi:10.1371/journal.pone.0226541)
Supplement: S1 Table — (DOCX) [file pone.0226541.s003.docx]

**S1 Table. The information of mixtures and the corresponding parameters.**

| Mixture number | Mixtures (A+B) | Toxicity ratio | C_A_ ^b^ | C_B_ ^b^ | $\log K_{f}^{A}$ | $\log K_{f}^{B}$ | Observed -log(EC_50M_) | Predicted -log(EC_50M_) |
| --- | --- | --- | --- | --- | --- | --- | --- | --- |
| 1 | Fe^3+^-Co^2+^ | 1:10 | 0.38 | 0.62 | 2.26 | 11.66 | 4.14 | 4.06 |
| 2 ^a^ |  | 1:10^0.5^ | 0.66 | 0.34 | 2.60 | 9.98 | 3.53 | 4.06 |
| 3 |  | 1:1 | 0.86 | 0.14 | 2.77 | 7.45 | 3.67 | 3.86 |
| 4 |  | 10^0.5^:1 | 0.95 | 0.05 | 2.83 | 4.45 | 3.63 | 3.78 |
| 5 |  | 10:1 | 0.98 | 0.02 | 2.85 | 1.32 | 3.71 | 3.72 |
| 6 ^a^ | Fe^3+^-Cr^3+^ | 1:10 | 0.10 | 0.90 | 1.41 | 2.56 | 3.62 | 3.72 |
| 7 |  | 1:10^0.5^ | 0.25 | 0.75 | 2.00 | 2.46 | 3.73 | 3.86 |
| 8 |  | 1:1 | 0.52 | 0.48 | 2.45 | 2.21 | 3.90 | 3.82 |
| 9 |  | 10^0.5^:1 | 0.77 | 0.23 | 2.70 | 1.78 | 3.87 | 3.77 |
| 10 ^a^ |  | 10:1 | 0.91 | 0.09 | 2.80 | 1.22 | 3.75 | 3.77 |
| 11 | Fe^3+^-Zn^2+^ | 1:10 | 0.67 | 0.33 | 2.61 | 9.45 | 4.30 | 3.90 |
| 12 ^a^ |  | 1:10^0.5^ | 0.86 | 0.14 | 2.77 | 7.06 | 3.83 | 3.90 |
| 13 |  | 1:1 | 0.95 | 0.05 | 2.83 | 4.18 | 3.54 | 3.77 |
| 14 |  | 10^0.5^:1 | 0.98 | 0.02 | 2.85 | 1.12 | 3.68 | 3.71 |
| 15 |  | 10:1 | 1.00 | 0.00 | 2.86 | -1.94 | 3.73 | 3.66 |
| 16 | Fe^3+^-Cu^2+^ | 1:10 | 0.40 | 0.60 | 2.29 | 13.93 | 4.17 | 4.19 |
| 17 |  | 1:10^0.5^ | 0.68 | 0.32 | 2.62 | 11.82 | 4.12 | 4.04 |
| 18 ^a^ |  | 1:1 | 0.87 | 0.13 | 2.77 | 8.72 | 4.06 | 4.04 |
| 19 |  | 10^0.5^:1 | 0.96 | 0.04 | 2.83 | 5.07 | 3.78 | 3.80 |
| 20 |  | 10:1 | 0.99 | 0.01 | 2.85 | 1.30 | 3.83 | 3.72 |
| 21 | Co^2+^-Cr^3+^ | 1:10 | 0.02 | 0.98 | 1.50 | 12.41 | 4.15 | 4.02 |
| 22 |  | 1:10^0.5^ | 0.05 | 0.95 | 4.62 | 12.32 | 3.85 | 4.16 |
| 23 |  | 1:1 | 0.15 | 0.85 | 7.61 | 12.03 | 4.24 | 4.30 |
| 24 |  | 10^0.5^:1 | 0.36 | 0.64 | 10.10 | 11.27 | 4.20 | 4.43 |
| 25 ^a^ |  | 10:1 | 0.63 | 0.37 | 11.73 | 9.74 | 4.11 | 4.43 |
| 26 | Co^2+^-Zn^2+^ | 1:10 | 0.25 | 0.75 | 9.08 | 2.46 | 4.56 | 4.25 |
| 27 |  | 1:10^0.5^ | 0.51 | 0.49 | 11.11 | 2.22 | 4.48 | 4.36 |
| 28 |  | 1:1 | 0.77 | 0.23 | 12.27 | 1.79 | 4.65 | 4.43 |
| 29 |  | 10^0.5^:1 | 0.91 | 0.09 | 12.76 | 1.23 | 4.51 | 4.46 |
| 30 |  | 10:1 | 0.97 | 0.03 | 12.94 | 0.61 | 4.60 | 4.46 |
| 31 ^a^ | Co^2+^-Cu^2+^ | 1:10 | 0.10 | 0.90 | 6.46 | 15.33 | 4.37 | 4.46 |
| 32 |  | 1:10^0.5^ | 0.25 | 0.75 | 9.15 | 14.68 | 4.51 | 4.66 |
| 33 |  | 1:1 | 0.52 | 0.48 | 11.18 | 13.17 | 4.59 | 4.68 |
| 34 |  | 10^0.5^:1 | 0.78 | 0.22 | 12.31 | 10.56 | 4.44 | 4.64 |
| 35 |  | 10:1 | 0.92 | 0.08 | 12.77 | 7.24 | 4.33 | 4.59 |
| 36 | Cr^3+^-Zn^2+^ | 1:10 | 0.66 | 0.34 | 2.38 | 9.57 | 4.52 | 4.03 |
| 37 |  | 1:10^0.5^ | 0.86 | 0.14 | 2.53 | 7.21 | 4.2 | 4.01 |
| 38 ^a^ |  | 1:1 | 0.95 | 0.05 | 2.59 | 4.34 | 3.98 | 4.01 |
| 39 |  | 10^0.5^:1 | 0.98 | 0.02 | 2.61 | 1.29 | 3.76 | 3.92 |
| 40 |  | 10:1 | 0.99 | 0.01 | 2.62 | -1.76 | 3.76 | 3.87 |
| 41 | Cr^3+^-Cu^2+^ | 1:10 | 0.39 | 0.61 | 2.08 | 14.01 | 4.20 | 4.27 |
| 42 |  | 1:10^0.5^ | 0.66 | 0.34 | 2.39 | 11.97 | 4.16 | 4.18 |
| 43 ^a^ |  | 1:1 | 0.86 | 0.14 | 2.54 | 8.91 | 4.40 | 4.18 |
| 44 |  | 10^0.5^:1 | 0.95 | 0.05 | 2.59 | 5.28 | 3.76 | 4.00 |
| 45 |  | 10:1 | 0.98 | 0.02 | 2.61 | 1.51 | 3.65 | 3.92 |
| 46 | Zn^2+^-Cu^2+^ | 1:10 | 0.03 | 0.97 | 3.15 | 15.57 | 4.56 | 4.44 |
| 47 |  | 1:10^0.5^ | 0.09 | 0.91 | 6.05 | 15.35 | 4.61 | 4.54 |
| 48 |  | 1:1 | 0.25 | 0.75 | 8.69 | 14.71 | 4.66 | 4.60 |
| 49 |  | 10^0.5^:1 | 0.51 | 0.49 | 10.66 | 13.22 | 4.76 | 4.58 |
| 50 |  | 10:1 | 0.77 | 0.23 | 11.74 | 10.71 | 4.73 | 4.58 |

^a^The mixture was selected in the validation sets in developing QSAR model.

^b^C_A_ and C_B_ presented as the percentage of component in the mixtur(mol/L), the original concentration datas of components were presented in S2 Fig.
