# Supplementary material for: The mixture toxicity of heavy metals on Photobacterium phosphoreum and its modeling by ion characteristics-based QSAR
Source: PLoS One. 2019 Dec 19;14(12):e0226541. doi: 10.1371/journal.pone.0226541 (PMC6922345; doi:10.1371/journal.pone.0226541)
Supplement: S2 Table — (DOCX) [file pone.0226541.s004.docx]

**S2 Table. Used ion characteristic descriptors of test heavy metals.**

|  | r | Xm | Xm^2^r | IP | ΔIP | AN/*Δ*IP | ︱logKOH︱ | AW | σP | *Δ*E | Z | Z^2^/r | Z* | Z*^2^/r |
| --- | --- | --- | --- | --- | --- | --- | --- | --- | --- | --- | --- | --- | --- | --- |
| Cu^2+^ | 0.73 | 1.9 | 2.64 | 20.29 | 12.55 | 2.31 | 8.0 | 63.54 | 0.104 | 0.16 | 1.39 | 2.64 | 14.25 | 282.031 |
| Co^2+^ | 0.75 | 1.88 | 2.65 | 17.08 | 9.18 | 2.94 | 9.7 | 58.93 | 0.13 | 0.28 | 1.41 | 2.65 | 12.95 | 204.515 |
| Zn^2+^ | 0.75 | 1.65 | 2.04 | 17.96 | 8.57 | 3.50 | 9.0 | 65.37 | 0.115 | 0.76 | 1.24 | 2.04 | 14.9 | 267.482 |
| Fe^3+^ | 0.65 | 1.83 | 2.18 | 30.65 | 14.44 | 1.80 | 2.2 | 55.847 | 0.103 | 0.77 | 3.00 | 13.85 | 12.65 | 238.84 |
| Cr^3+^ | 0.62 | 1.66 | 1.71 | 30.96 | 14.46 | 1.66 | 4.0 | 51.996 | 0.107 | 0.41 | 1.03 | 1.71 | 11.35 | 198.188 |
