# Supplementary material for: The mixture toxicity of heavy metals on Photobacterium phosphoreum and its modeling by ion characteristics-based QSAR
Source: PLoS One. 2019 Dec 19;14(12):e0226541. doi: 10.1371/journal.pone.0226541 (PMC6922345; doi:10.1371/journal.pone.0226541)
Supplement: S3 Table — (DOCX) [file pone.0226541.s005.docx]

**S3 Table. The ion characteristic descriptors of mixtures that was calculated on the basis of Eq.4.**

| Mix^a^ | r^m^ | Xm^m^ | Xm^2^r^m^ | IP^m^ | ΔIP^m^ | (AN/*Δ*IP) ^m^ | ︱logKOH︱^m^ | AW^m^ | σP^m^ | *Δ*E^m^ | Z^m^ | (Z^2^/r) ^m^ | (Z*)^m^ | (Z*^2^/r) ^m^ |
| --- | --- | --- | --- | --- | --- | --- | --- | --- | --- | --- | --- | --- | --- | --- |
| Mix-1 | 0.71 | 1.86 | 2.47 | 22.26 | 11.19 | 2.51 | 6.84 | 57.75 | 0.12 | 0.47 | 2.02 | 6.92 | 12.84 | 217.61 |
| Mix-2 | 0.68 | 1.85 | 2.34 | 26.02 | 12.65 | 2.19 | 4.76 | 56.90 | 0.11 | 0.60 | 2.46 | 10.02 | 12.75 | 227.11 |
| Mix-3 | 0.66 | 1.84 | 2.24 | 28.76 | 13.71 | 1.96 | 3.25 | 56.28 | 0.11 | 0.70 | 2.78 | 12.29 | 12.69 | 234.05 |
| Mix-4 | 0.65 | 1.83 | 2.20 | 30.00 | 14.19 | 1.85 | 2.56 | 56.00 | 0.10 | 0.75 | 2.92 | 13.31 | 12.66 | 237.18 |
| Mix-5 | 0.65 | 1.83 | 2.18 | 30.44 | 14.36 | 1.82 | 2.32 | 55.90 | 0.10 | 0.76 | 2.98 | 13.67 | 12.65 | 238.29 |
| Mix-6 | 0.62 | 1.68 | 1.75 | 30.93 | 14.46 | 1.67 | 3.83 | 52.37 | 0.11 | 0.44 | 1.22 | 2.88 | 11.48 | 202.11 |
| Mix-7 | 0.63 | 1.70 | 1.83 | 30.88 | 14.45 | 1.69 | 3.55 | 52.96 | 0.11 | 0.50 | 1.52 | 4.74 | 11.67 | 208.35 |
| Mix-8 | 0.64 | 1.75 | 1.95 | 30.80 | 14.45 | 1.73 | 3.07 | 53.98 | 0.10 | 0.60 | 2.05 | 7.98 | 12.02 | 219.17 |
| Mix-9 | 0.64 | 1.79 | 2.07 | 30.72 | 14.45 | 1.77 | 2.61 | 54.97 | 0.10 | 0.69 | 2.55 | 11.10 | 12.36 | 229.63 |
| Mix-10 | 0.65 | 1.82 | 2.14 | 30.68 | 14.45 | 1.79 | 2.35 | 55.52 | 0.10 | 0.74 | 2.83 | 12.81 | 12.54 | 235.36 |
| Mix-11 | 0.68 | 1.77 | 2.13 | 26.47 | 12.51 | 2.36 | 4.44 | 58.98 | 0.11 | 0.77 | 2.42 | 9.96 | 13.39 | 248.27 |
| Mix-12 | 0.66 | 1.81 | 2.16 | 28.93 | 13.65 | 2.03 | 3.12 | 57.14 | 0.10 | 0.77 | 2.76 | 12.25 | 12.96 | 242.73 |
| Mix-13 | 0.65 | 1.82 | 2.17 | 30.06 | 14.17 | 1.88 | 2.52 | 56.29 | 0.10 | 0.77 | 2.92 | 13.30 | 12.76 | 240.18 |
| Mix-14 | 0.65 | 1.83 | 2.17 | 30.46 | 14.36 | 1.83 | 2.30 | 55.99 | 0.10 | 0.77 | 2.97 | 13.67 | 12.68 | 239.27 |
| Mix-15 | 0.65 | 1.83 | 2.18 | 30.59 | 14.42 | 1.81 | 2.23 | 55.89 | 0.10 | 0.77 | 2.99 | 13.79 | 12.66 | 238.98 |
| Mix-16 | 0.70 | 1.87 | 2.45 | 24.46 | 13.31 | 2.10 | 5.67 | 60.45 | 0.10 | 0.41 | 2.04 | 7.15 | 13.61 | 264.66 |
| Mix-17 | 0.68 | 1.85 | 2.32 | 27.31 | 13.84 | 1.96 | 4.07 | 58.33 | 0.10 | 0.57 | 2.48 | 10.24 | 13.17 | 252.76 |
| Mix-18 | 0.66 | 1.84 | 2.24 | 29.31 | 14.20 | 1.87 | 2.95 | 56.84 | 0.10 | 0.69 | 2.79 | 12.40 | 12.86 | 244.43 |
| Mix-19 | 0.65 | 1.83 | 2.20 | 30.19 | 14.36 | 1.82 | 2.46 | 56.19 | 0.10 | 0.74 | 2.93 | 13.35 | 12.72 | 240.76 |
| Mix-20 | 0.65 | 1.83 | 2.18 | 30.50 | 14.42 | 1.81 | 2.28 | 55.96 | 0.10 | 0.76 | 2.98 | 13.69 | 12.67 | 239.47 |
| Mix-21 | 0.75 | 1.65 | 2.05 | 17.95 | 8.58 | 3.49 | 9.01 | 65.26 | 0.12 | 0.75 | 1.24 | 2.05 | 14.87 | 266.41 |
| Mix-22 | 0.75 | 1.66 | 2.07 | 17.92 | 8.60 | 3.47 | 9.04 | 65.04 | 0.12 | 0.74 | 1.25 | 2.07 | 14.80 | 264.25 |
| Mix-23 | 0.75 | 1.68 | 2.13 | 17.83 | 8.66 | 3.42 | 9.10 | 64.42 | 0.12 | 0.69 | 1.26 | 2.13 | 14.61 | 258.20 |
| Mix-24 | 0.75 | 1.73 | 2.26 | 17.65 | 8.79 | 3.30 | 9.25 | 63.08 | 0.12 | 0.59 | 1.30 | 2.26 | 14.21 | 245.05 |
| Mix-25 | 0.75 | 1.80 | 2.43 | 17.41 | 8.96 | 3.15 | 9.44 | 61.29 | 0.12 | 0.46 | 1.35 | 2.43 | 13.66 | 227.58 |
| Mix-26 | 0.65 | 1.71 | 1.94 | 27.51 | 13.15 | 1.98 | 5.42 | 53.72 | 0.11 | 0.38 | 1.12 | 1.94 | 11.75 | 199.76 |
| Mix-27 | 0.69 | 1.77 | 2.19 | 23.91 | 11.78 | 2.31 | 6.90 | 55.52 | 0.12 | 0.34 | 1.22 | 2.19 | 12.16 | 201.40 |
| Mix-28 | 0.72 | 1.83 | 2.43 | 20.31 | 10.41 | 2.64 | 8.38 | 57.32 | 0.12 | 0.31 | 1.32 | 2.43 | 12.58 | 203.04 |
| Mix-29 | 0.74 | 1.86 | 2.57 | 18.28 | 9.64 | 2.83 | 9.21 | 58.33 | 0.13 | 0.29 | 1.38 | 2.57 | 12.81 | 203.97 |
| Mix-30 | 0.75 | 1.87 | 2.62 | 17.49 | 9.34 | 2.90 | 9.53 | 58.73 | 0.13 | 0.28 | 1.40 | 2.62 | 12.90 | 204.33 |
| Mix-31 | 0.73 | 1.90 | 2.64 | 19.98 | 12.22 | 2.37 | 8.17 | 63.09 | 0.11 | 0.17 | 1.39 | 2.64 | 14.12 | 274.40 |
| Mix-32 | 0.74 | 1.89 | 2.64 | 19.48 | 11.70 | 2.47 | 8.43 | 62.37 | 0.11 | 0.19 | 1.39 | 2.64 | 13.92 | 262.32 |
| Mix-33 | 0.74 | 1.89 | 2.64 | 18.62 | 10.80 | 2.64 | 8.89 | 61.13 | 0.12 | 0.22 | 1.40 | 2.65 | 13.57 | 241.58 |
| Mix-34 | 0.75 | 1.88 | 2.65 | 17.80 | 9.93 | 2.80 | 9.32 | 59.96 | 0.12 | 0.25 | 1.40 | 2.65 | 13.24 | 221.77 |
| Mix-35 | 0.75 | 1.88 | 2.65 | 17.35 | 9.47 | 2.89 | 9.56 | 59.32 | 0.13 | 0.27 | 1.41 | 2.65 | 13.06 | 211.02 |
| Mix-36 | 0.66 | 1.66 | 1.82 | 26.49 | 12.43 | 2.29 | 5.72 | 56.59 | 0.11 | 0.53 | 1.10 | 1.82 | 12.57 | 222.00 |
| Mix-37 | 0.64 | 1.66 | 1.76 | 29.09 | 13.61 | 1.92 | 4.72 | 53.92 | 0.11 | 0.46 | 1.06 | 1.76 | 11.86 | 208.13 |
| Mix-38 | 0.63 | 1.66 | 1.73 | 30.31 | 14.16 | 1.75 | 4.25 | 52.66 | 0.11 | 0.43 | 1.04 | 1.73 | 11.53 | 201.64 |
| Mix-39 | 0.62 | 1.66 | 1.71 | 30.75 | 14.36 | 1.69 | 4.08 | 52.21 | 0.11 | 0.42 | 1.03 | 1.72 | 11.41 | 199.30 |
| Mix-40 | 0.62 | 1.66 | 1.71 | 30.89 | 14.43 | 1.67 | 4.03 | 52.07 | 0.11 | 0.41 | 1.03 | 1.71 | 11.37 | 198.55 |
| Mix-41 | 0.69 | 1.81 | 2.28 | 24.42 | 13.29 | 2.06 | 6.45 | 59.07 | 0.11 | 0.26 | 1.25 | 2.28 | 13.13 | 249.60 |
| Mix-42 | 0.66 | 1.74 | 2.02 | 27.37 | 13.82 | 1.88 | 5.35 | 55.88 | 0.11 | 0.33 | 1.15 | 2.02 | 12.33 | 226.40 |
| Mix-43 | 0.64 | 1.69 | 1.84 | 29.50 | 14.20 | 1.75 | 4.55 | 53.58 | 0.11 | 0.38 | 1.08 | 1.84 | 11.75 | 209.66 |
| Mix-44 | 0.63 | 1.67 | 1.75 | 30.46 | 14.37 | 1.69 | 4.19 | 52.54 | 0.11 | 0.40 | 1.05 | 1.75 | 11.49 | 202.14 |
| Mix-45 | 0.62 | 1.66 | 1.72 | 30.79 | 14.43 | 1.67 | 4.06 | 52.18 | 0.11 | 0.41 | 1.04 | 1.72 | 11.40 | 199.50 |
| Mix-46 | 0.73 | 1.89 | 2.62 | 20.22 | 12.43 | 2.35 | 8.03 | 63.60 | 0.10 | 0.18 | 1.38 | 2.62 | 14.27 | 281.57 |
| Mix-47 | 0.73 | 1.88 | 2.58 | 20.07 | 12.18 | 2.42 | 8.09 | 63.71 | 0.11 | 0.22 | 1.37 | 2.58 | 14.31 | 280.67 |
| Mix-48 | 0.73 | 1.84 | 2.49 | 19.71 | 11.57 | 2.61 | 8.25 | 63.99 | 0.11 | 0.31 | 1.35 | 2.49 | 14.41 | 278.42 |
| Mix-49 | 0.74 | 1.77 | 2.33 | 19.10 | 10.51 | 2.92 | 8.51 | 64.48 | 0.11 | 0.47 | 1.31 | 2.33 | 14.58 | 274.55 |
| Mix-50 | 0.75 | 1.71 | 2.18 | 18.50 | 9.50 | 3.22 | 8.77 | 64.94 | 0.11 | 0.62 | 1.27 | 2.18 | 14.75 | 270.86 |
